# Ion Mobility Studies on the Negative Ion-Molecule Chemistry of Isoflurane and Enflurane

**DOI:** 10.1007/s13361-017-1616-0

**Published:** 2017-02-21

**Authors:** Ramón González-Méndez, Peter Watts, David C. Howse, Immacolata Procino, Henry McIntyre, Chris A. Mayhew

**Affiliations:** 1 School of Physics and Astronomy, University of Birmingham, Edgbaston, Birmingham, B15 2TT UK; 2 Smiths Detection, Century House, Maylands Avenue, Hemel Hempstead, Hertfordshire HP2 7DE UK; 3 Institut für Atemgasanalytik, Leopold-Franzens-Universität Innsbruck, Rathausplatz 4, A-6850 Dornbirn, Austria

**Keywords:** Ion mobility spectrometry, IMS-MS, Isoflurane, Enflurane, Ion-molecule reactions

## Abstract

In the present work we present an investigation of the negative ion-molecule chemistry of the anaesthetics isoflurane, ISOF, and enflurane, ENF, in an ion mobility spectrometry/mass spectrometry (IMS/MS), in both air and nitrogen. Hexachloroethane (HCE) was introduced in both air and nitrogen to produce Cl^–^ as a reactant ion. This study was undertaken owing to uncertainties in the chemical processes, which lead to the cluster ions reported in other work (Eiceman et al. Anal. Chem. 61, 1093–1099, 1). In particular for ISOF the product ion observed was ISOF.Cl^–^, and it was suggested that the Cl^–^ was formed by dissociative electron attachment (DEA) although there was mention of a chlorine containing contaminant. We show in this study that ISOF and ENF do not produce Cl^–^ in an IMS system either by capture of free electrons or reaction with O_2_^–^. This demonstrates that the Cl^–^ containing ions, reported in the earlier study, must have been the result of a chlorine containing contaminant as suggested. The failure of ISOF and ENF to undergo DEA was initially surprising given the high calculated electron affinities, but further calculations showed that this was a result of the large positive vertical attachment energies (VAEs). This experimental work has been supported by electronic structure calculations at the B3LYP level, and is consistent with those obtained in a crossed electron-molecular beam two sector field mass spectrometer. An unusual observation is that the monomer complexes of ISOF and ENF with O_2_^–^ are relatively unstable compared with the dimer complexes.

**Electronic supplementary material:**

The online version of this article (doi:10.1007/s13361-017-1616-0) contains supplementary material, which is available to authorized users.

**Graphical Abstract:**
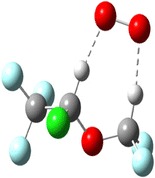
ᅟ

## Introduction

Isoflurane (ISOF) and enflurane (ENF) are volatile halogenated ethers that are used as anaesthetics in human medicine, although their use is starting to decline and being replaced with sevoflurane. The use of ion mobility spectrometry/mass spectrometry (IMS/MS) to detect and monitor ISOF, CF_3_CHClOCHF_2_, and ENF, CHF_2_OCF_2_CHFCl has been reported previously [1], and a device designed for that purpose has been patented [2]. ISOF has also been proposed as a chemical standard for calibrating IMS systems [3]. Cluster ions such as ISOF.Cl^–^ and ENF.O_2_^–^ were reported and it was suggested that the Cl^–^ was formed by dissociative electron attachment (DEA), although there was mention of a chlorine containing contaminant [1]. In this paper, we report a reinvestigation of this earlier work to clarify the ion chemistry involved in an IMS system. As will be seen, no Cl^–^ containing ions were observed in either air or nitrogen with ISOF and ENF. Hexachloroethane (HCE) was therefore introduced to produce Cl^–^ ions in an attempt to replicate the earlier work.

HCE has been used as a dopant in IMS for the detection of explosives [4–8]; its chemistry, however, has not been studied in depth, so a secondary aim of this present paper is a report of the results of our study of HCE.

The experimental work presented here has been supported by electronic structure calculations using the B3LYP functional and the 6-31 + G(d,p) basis set.

## Experimental

### Ion Mobility Spectrometry-Mass Spectrometry (IMS/MS)

IMS is a gas-phase analytical instrument used to temporally separate reactant and product ions in a drift tube according to their mobility [9]. The IMS/MS system used in this study has been described elsewhere [10–14]. In brief, the instrument consists of two drift tube regions, each 10 cm in length. The first, the reaction region containing a cylindrical radioactive ion source (nominal 10 mCi ^63^Ni foil), is physically separated from the second region, the drift region, by a Bradbury-Nielson (B-N) gate. A forward flow of the buffer gas flows through the radioactive source and into the glass jacket towards the B-N grid, and in this forward flow the analyte to be investigated is introduced. A contraflow of the same buffer gas is introduced through apertures near to a Faraday plate (FP). Typical forward and contraflows are 0.4 L min^–1^ and 0.8 L min^–1^ (at slightly above the ambient atmospheric pressure and room temperature), respectively. These flows are controlled by mass flow controllers (Alicat, ±1% accuracy). The two flows are vented out of the drift tube through holes in the B-N ring. The drift tube’s pressure is measured with a strain gauge absolute pressure sensor (Edwards, model ASG 2000). A thermocouple is used to monitor the temperature of the buffer gas near to the exhaust region. The temperature of the drift tube is electronically controlled at a constant temperature of 30 ± 1 °C to avoid the need to compensate for changes in ambient temperature. An electric field along the axis of the drift tube is set at 200 V∙cm^–1^ by applying a suitable voltage gradient across the whole of the drift tube.

The FP is protected by a screen grid to shield it from the electric field produced by the oncoming ion swarm. At the center of the FP there is a 0.07 mm pinhole, separating the IMS from the lower pressure quadrupole mass spectrometer region. The product ions are separated according to their *m*/*z* values using quadrupole mass filter and detected using a secondary electron multiplier. For this identification of the *m/z* values, the B-N grid in the drift tube is kept open in order to maximize ion signal intensity.

To obtain ion mobility spectra, the B-N gate is used to pulse reactant and product ions generated in the reaction region into the drift region at a frequency of 25 Hz and a pulse width of 600 μs (600 μs was necessary because at shorter pulse widths the ion signals associated with isoflurane and enflurane were significantly weaker, presumably owing to the transit times of the product ions through the B-N grid). Mobility spectra were acquired by means of intentionaly written software using Labview [14]. Total ion mobility spectra were acquired using the FP. Tuned ion mobility spectra were obtained by sampling ions through the FP and then allowing a specific *m*/*z* through the mass filter. The tuned ion mobility spectra were used to verify contributions of product ions to the individual peaks in the total ion mobility spectra.

### Procedures and Chemicals

Isoflurane and hexachloroethane were purchased from Sigma Aldrich (UK), both with stated purities of 99%. Enflurane was purchased from Fluorchem Ltd. (UK) with a stated purity of 97%. All chemicals were used without further purification. At room temperature isoflurane and enflurane are liquids and hexachloroethane is a white granulated solid. For the liquid samples, typically 50 μL were spotted onto cotton placed inside a glass syringe (Weber Scientific, UK), which was inserted through a septum into the forward flow. A syringe driver (Cole Palmer 74900 series; IL, USA) was used to introduce the compound into the forward flow at a constant rate. For hexachloroethane, a few mg were deposited into a glass vial sealed with a PTFE septum (Thames Restek, Bellefonte, PA, USA) through which the forward gas flowed.

Zero air grade and pure nitrogen (oxygen free and 99.998% minimum nitrogen) carrier gases used for this experiment were purchased from BOC Gases (UK). Prior to entering the reaction region, all carrier gases were passed through moisture and hydrocarbon traps (Supelco 23991 and Agilent BHT-4, respectively).

### DFT Calculations

These were conducted using Gaussian09W and GaussView05 for Windows [15]. All calculations used the B3LYP hybrid functional and (unless stated otherwise) the 6-31 + G(d,p) basis set. We have found this combination to give fair agreement with published values of the adiabatic electron affinities of species such as Cl and O_2_ and 1,3,5-trinitrobenzene and reactions such as OH^–^ with H^+^ and Cl^–^ with H^+^ [16]. Stable species were characterized by the absence of an imaginary frequency. Adiabatic electron affinities (AEAs) were determined by calculating the total energy of an anion at its optimized geometry and subtracting from this the total energy of the neutral at its optimized geometry. Vertical attachment energies (VAE) correspond to the change in energy on attachment of an electron to the ground state of a molecule without any change of nuclear geometry. These were determined by doing a frequency calculation after placing a negative charge on the ground state geometry of the neutral and then subtracting the computed total energy of the neutral from that of the anion.

## Results and Discussion

### Electron Attachment

*ISOF* Despite both electron attachment (EA) and dissociative electron attachment (DEA) being calculated thermodynamically favorable, adiabatic EA 1.76 eV (170 kJ mol^–1^) and DEA to yield Cl^–^ ΔH_298_ = –65 kJ mol^–1^ and ΔG_298_ = –106 kJ mol^–1^, neither was observed with near-thermal electrons in nitrogen. This suggests that the VAE is positive. As calculated VAEs, given in Table 1, and adiabatic EAs are basis set-dependent, the influence of basis set was briefly investigated using the B3LYP functional [17–19]. A representation of the structure of the anion ISOF^-^ derived from DFT calculations is shown in Supplementary Figure S1.

**Table 1:** Dependence of EA and VAE in kJ∙mol^–1^ upon Basis Set Using the B3LYP Functional at 298 K. In the VAE Column the Figures in Parentheses are the Number of Imaginary Frequencies Observed

Basis set	EA kJ∙mol^–1^	VAE kJ∙mol^–1^
6-31 + G(d,p)	170	+60 (4)
6-31++G(d,p)	170	+39 (3)
6-31 + G(3df,2p)	154	+60 (3)
6-31++G(3df,2p)	154	+45 (1)
6-31 + G(2d,2p)	161	+62 (3)
6-311++G(3df,2p)	156	+45 (1)
6-31G(d,p)	109	+182 (3)

The influence of the basis set shows a different pattern for the EA and VAE. The most important feature of the basis set is to have a diffuse function – increasing this from + to ++ has no effect upon the EA but causes a decrease in the VAE. Increasing the polarization functions from (d,p) to (3df,2p) has a small effect upon the EA but not upon the VAE. Going from 6-31 to 6-311 has no effect upon either, at least with the diffuse and polarization functions used. Overall, so long as both polarization and diffuse functions are included, the calculated EA and VAE are of sufficient accuracy to allow the identification of trends and to arrive at an understanding of the experimental observations.

Inspection of the imaginary frequencies in the VAE calculations showed that even when there were multiple imaginary frequencies, one was always dominant (i.e., was considerably more intense than other imaginary frequencies), and was associated with stretching of the C–Cl bond. IRC Intrinsic reaction coordinate (IRC) calculations were used to follow the C–Cl stretch and showed that the initial structure associated with the VAE relaxed to the structure found in the calculation of the adiabatic EA. It is suggested that the non-observation of attachment of thermal electrons is the result of a VAE of ca. +60 kJ∙mol^–1^ (0.62 eV). This is in excellent agreement with a resonance of 0.6 eV leading to Cl^–^ observed in a recent study of DEA of ENF, ISOF, and HCE in a crossed electron-molecular beam two sector field mass spectrometer [20].

**Figure 1:**
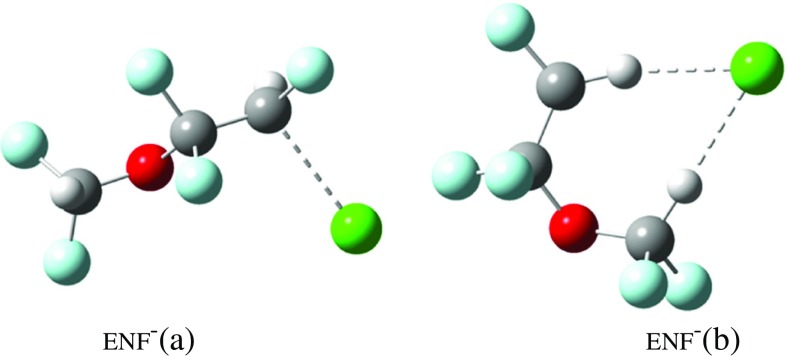
Structure of two possible negative ENF ions obtained from DFT calculations

*ENF* ENF is a little more complicated than ISOF as calculations show that there are two stable negative ions denoted ENF^–^(a) and ENF^–^(b) in the structures (Figure 1). ENF has an effective EA of 104 kJ∙mol^–1^ (1.08 eV) when forming ENF^–^(a) and an effective EA of 146 kJ∙mol^–1^ (1.51 eV) when forming ENF^–^(b). DEA is thermodynamically favorable with ΔH_298_ = –65 kJ∙mol^–1^ and ΔG_298_ = –106 kJ∙mol^–1^. However, as seen earlier for ISOF, neither EA nor DEA is observed. The VAE is calculated (using 6-31 + G(d,p)) to be +74 kJ∙mol^–1^ (0.77 eV) with four imaginary frequencies. An IRC calculation on this species shows it relaxing to give ENF^-^(a).

Attempts were made to find a transition state between ENF^–^(a) and ENF^–^(b) but these were unsuccessful. Various relaxed scans of bond lengths and dihedral angles were investigated on the structure of ENF^–^(a) and numerous stable configurations differing by only a few kJ∙mol^–1^ were found, and when one approached close to ENF^–^(b) it just flicked over with no discernible transition state.

### Reactions in Air

*ISOF* Although thermodynamically feasible (see Table 2), neither electron transfer nor dissociative electron transfer from O_2_^–^ is observed. Neither is proton abstraction; only complexation with O_2_^-^ occurs.

**Table 2:** ΔHs and ΔGs for the Possible Reactions of ISOF in Air. DFT Calculations Were Performed Using the B3LYP Functional and the 6-31 + G (d,p) Basis Set

Reactants	Ionic products	ΔH_298_ kJ∙mol^–1^	ΔG_298_ kJ∙mol^–1^
ISOF + e	ISOF^–^	–170	–183
	CHF_2_OCHCF_3_ + Cl^–^	–65	–106
ISOF + O_2_^–^	ISOF^–^ + O_2_	–110	–124
	ISOF^–^.O_2_	–146	–111
ISOF^–^.O_2_ + ISOF	ISOF_2_^–^.O_2_	–92	–51

**Figure 2:**
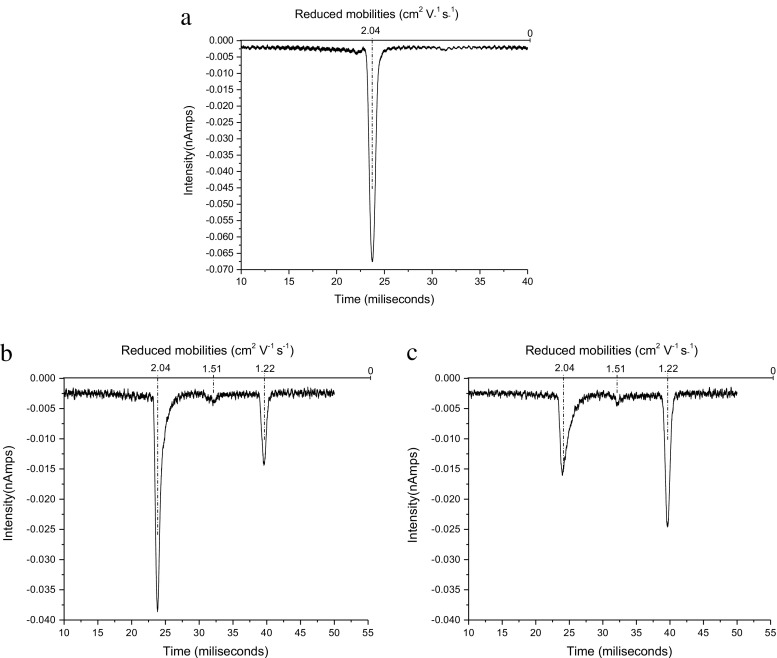
IMS spectra showing (**a**) the air RIP and air doped with ISOF showing the air RIP, ISOF.O_2_^–^ and (ISOF)_2_.O_2_^–^ peaks for two ISOF concentrations (**b**) lower and (**c**) higher

The air reactant ion peak (RIP) is initially sharp and reasonably symmetrical (see Figure 2a). It is appreciated that the air RIP is a complex with the O_2_^–^ ions being complexed to varying degrees with O_2_, H_2_O, and CO_2_, and that the degree of complexation varies during the migration of the ions down the reaction region (for a detailed description of the air RIP see Hayhurst et al. [21] and Watts [22]). But as only complexation with O_2_^–^ is observed, discussion of the potential complexation with other negative RIP ions will not be considered further.

**Figure 3:**
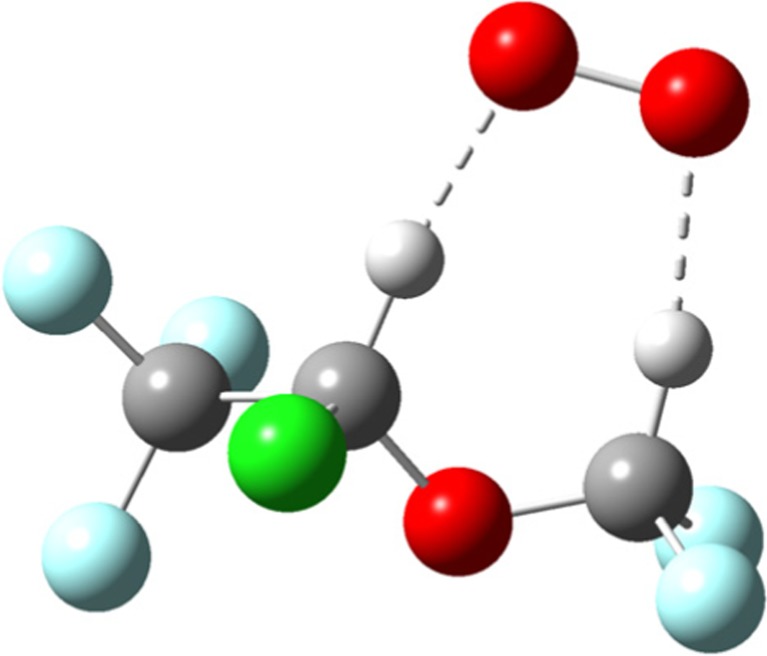
Structure for the stable monomer ISOF.O_2_^–^ from DFT calculations

Addition of sufficient ISOF to decrease the RIP by 50% shows a good dimer peak ((ISOF)_2_.O_2_^–^), and a small monomer (ISOF.O_2_^–^) [Neither structural information nor charge distribution is implied when identifying an ion in this form]. (see Figure 2b and c). Broadening of the RIP on the low mobility side is observed. This is consistent with an unstable monomer complex being formed, which can either rearrange to give a stable monomer or which can decompose to give O_2_^–^ related ions to broaden the RIP. The monomer can react with more ISOF to give a stable dimer (ISOF)_2_.O_2_^–^. DFT calculations show that there is only one stable monomer, the structure of which is shown below (Figure 3) and the energetics provided in Table 2.

A search for less stable complex(es) of ISOF and O_2_^–^ was made. No minima were found, suggesting that the potential energy surface is relatively flat.

The number of possible structures of the dimer anion is greater than the two shown in Supplementary Figure S2 as both ISOF and ENF are chiral and are sold as racemic mixtures and, thus, for any possible dimer structure there are two substructures either both molecules having the same configuration (i.e., R + R or S + S) or different configurations (i.e., R + S). A selection has been investigated, but as each ISOF is acting as a bidentate ligand (to give slightly puckered 7-membered rings) in the dimer they all have similar energies.

*ENF* As with ISOF, although thermodynamically feasible, neither electron transfer nor dissociative electron transfer from O_2_^–^ is observed, see Table 3.

**Table 3:** ΔHs and ΔGs for the Reaction of ENF in air. DFT Calculations Were Performed Using the B3LYP Functional and the 6-31 + G (d,p) Basis Set

Reactants	Products	ΔH_298_ kJ∙mol^–1^	ΔG_298_ kJ∙mol^–1^
ENF + e	ENF^–^(a)	–104	–115
	ENF^–^(b)	–146	–154
	CHF_2_OCF_2_CHF + Cl^–^	–52	–93
ENF + O_2_^-^	ENF–(a) + O_2_	–44	–55
	ENF–(b) + O_2_	–86	–94
	ENF.O_2_–(a)	–87	–45
	ENF.O_2_–(b)	–96	–62
	ENF.O_2_–(c)	–138	–101
ENF.O_2_^–^(c) + ENF	ENF_2_.O_2_–(a)	–83	–41
	ENF_2_.O_2_–(b)	–56	–20

**Figure 4:**
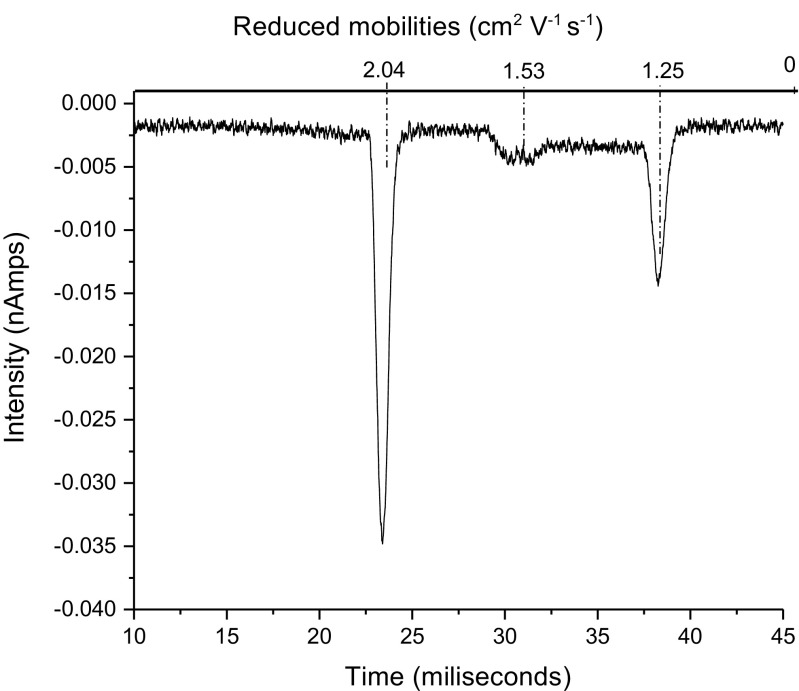
IMS spectrum after adding sufficient ENF to decrease the air RIP by about 50%

Addition of sufficient ENF to reduce the air RIP by ca. 50% gave a good dimer peak bridging (i.e., with an elevated baseline indicating decomposition of the dimer to the monomer, to a small monomer). Unlike with ISOF, no broadening of the RIP is observed (see Figure 4).

In contrast to ISOF, three stable structures for the monomer ENF.O_2_^–^ have been found and are shown in Supplementary Figure S3. Whilst transition states (TS) between these three ENF.O_2_^–^ monomer structures have yet to be found, it is likely that the TS energies will be small and that the energies for the formation of the complexes ENF.O_2_^—^[(a) and (b)] will be sufficient to overcome them, leading to ENF.O_2_^–^(c) being the only stable monomer observed—this has a similar structure to that shown for ISOF.O_2_^–^ in Figure 3. As the monomer peak is small and the RIP has not broadened, this suggests that initial complexes of ENF.O_2_^–^ are so unstable that they decompose sufficiently rapidly that the reformed O_2_^–^ ions are encompassed in the RIP.

The formation and structures of the dimer (ENF)_2_.O_2_^–^ is more complex than was found for the case with ISOF. Several structures (many if the formation of a dimer from ENF.O_2_^–^[(a) and (b)] are considered) of a dimer from ENF.O_2_^–^(c) are possible, but only two have negative ∆Gs of formation. These are shown in Supplementary Figure S4.

The sharp dimer peak at K_o_ = 1.25 cm^2^∙V^–1^∙s^–1^ shown in Figure 4 is assigned to (ENF)_2_.O_2_^–^(a). The unstable dimer anion causing the bridging between the monomer and dimer anions and the broadening of the monomer is therefore assigned as (ENF)_2_.O_2_^–^(b). The lower stability of the cyclic complexes containing O_2_^–^ compared with the corresponding complexes of ISOF is to be expected as the former forms an 8-membered ring whereas ISOF only requires a 7-membered ring.

### Reactions in Air Doped with Hexachloroethane (HCE)

**Figure 5:**
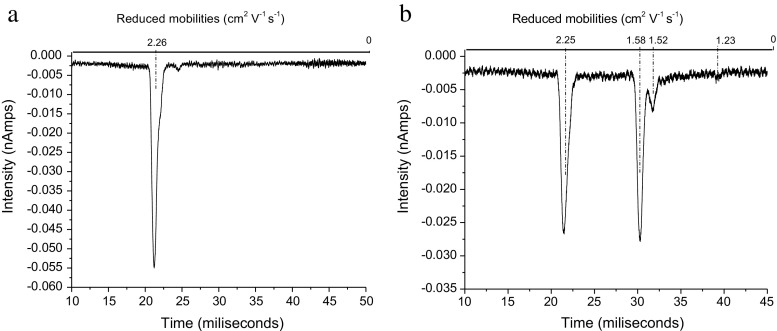
IMS spectra of (a) air doped with HCE and (b) after introducing a small amount of ISOF

On addition of HCE to an air system, a more mobile RIP is produced consisting of a sharp peak (Cl^–^ related ions) with a trace of a shoulder on the less mobile side (Cl_2_^–^) – see Figure 5a. A similar RIP is also seen on introduction of HCE into a nitrogen system. This is to be expected as the calculated VAE is –33 kJ mol^–1^ and the overall thermodynamics are favorable – see Table 4.

**Table 4:** ΔHs and ΔGs for the DEA of HCE. DFT Calculations Were Performed Using the B3LYP Functional and the 6-31 + G (d,p) Basis Set

Reactants	Ionic products	ΔH_298_ kJ∙mol^–1^	ΔG_298_ kJ∙mol^–1^
HCE + e	Cl^–^	–122	–170
	Cl_2_^–^	–215	–275
HCE + O_2_^–^	Cl^–^	–62	–110
	Cl_2_^–^	–152	–216

*ISOF* Addition of ISOF to an air system doped with HCE (similar to the one shown in Figure 5a), shows two peaks, ISOF.Cl^–^ and ISOF.Cl_2_^–^ together with a suggestion of an unstable dimer (ISOF)_2_.Cl^–^, (Figure 5b. ISOF.Cl_2_^–^ does not form a dimer. These observations are in agreement with the thermodynamics given in Table 5. The structures of the Cl^–^ and Cl_2_^–^ complexes with ISOF are similar to those with O_2_^–^ when the O_2_^–^ is acting as a bidentate ligand (i.e., forms a ring).

**Table 5:** ΔHs and ΔGs for the Possible Reactions of ISOF and HCE in Air. DFT Calculations were Performed Using the B3LYP Functional and the 6-31 + G (d,p) Basis Set

Reactants	Ionic products	ΔH_298_ kJ∙mol^–1^	ΔG_298_ kJ∙mol^–1^
ISOF + Cl^–^	ISOF.Cl^–^	–108	–79
ISOF.Cl^-^ + ISOF	ISOF_2_.Cl^–^	–74	–40
ISOF + Cl_2_^–^	ISOF.Cl_2_^–^	–75	–44
ISOF.Cl_2_^-^ + ISOF	ISOF_2_.Cl_2_^–^	–58	–2

**Figure 6:**
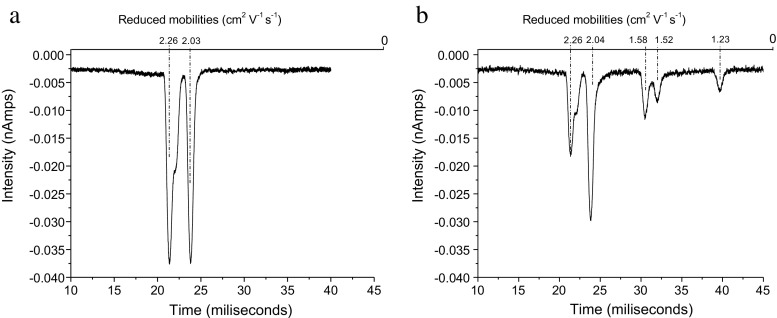
IMS spectra of air doped with enough HCE to (**a**) have similar intensities of air RIP and HCE RIP, (**b**) to have similar amounts of air RIP and HCE RIP with the addition of sufficient ISOF to decrease the HCE RIP by about 50%

It was observed that the air system was much less sensitive to ISOF than was the HCE system. In order to verify this, sufficient HCE was introduced into an air system to give an approximately 50/50 mix of air and HCE RIPs, as seen in Figure 6a.

What is immediately apparent is that the Cl_2_^–^ contribution to the HCE RIP appears to be much greater than in Figure 2b and c. Addition of ISOF gives three product peaks ISOF.Cl^–^, ISOF.Cl_2_^–^, and (ISOF)_2_.O_2_^–^ (see Figure 6b), thus confirming the observation of the high sensitivity to ISOF in a doped system compared with an undoped system.

The suggestion that more Cl_2_^–^ is formed in a partially doped system is confirmed by the changing ratio of ISOF.Cl^–^ to ISOF.Cl_2_^–^ in Figure 5b and Figure 6b. Why should this be? On full doping, HCE dominates the electron capture but on partial doping oxygen competes successfully for electrons. The reaction of O_2_^–^ with HCE favors the production of Cl_2_^–^ over Cl^–^.Mass spectra data show that in the 50/50 system the ratio of Cl^-^/Cl_2_^–^ is ca. 3.5, whereas in a normally doped system (sufficient HCE to remove the air RIP) the ratio is ca. 5.2. Increasing the HCE concentration much higher leads to a ratio of ca. 19, similar to that in nitrogen. The ratio in nitrogen is insensitive to the HCE concentration.

As the DEA to yield Cl_2_^–^ is energetically more favorable than that yielding Cl^–^, it might be expected that it would be the dominant pathway. However, since DEA to yield Cl^–^ is energetically favorable and can occur with any conformation of HCE, it may be that the Cl_2_^–^ can only be formed when the chlorines in the HCE are in the unstable eclipsed conformation, thus accounting for its low abundance. It is suggested that as well as direct dissociative electron transfer (DET from O_2_^–^ to HCE to give a similar Cl^–^/Cl_2_^–^ as in DEA, a transient complex of HCE and O_2_^–^ is formed in which the chlorines are in a more eclipsed conformation, thus leading to more Cl_2_^–^ being formed.

**Figure 7:**
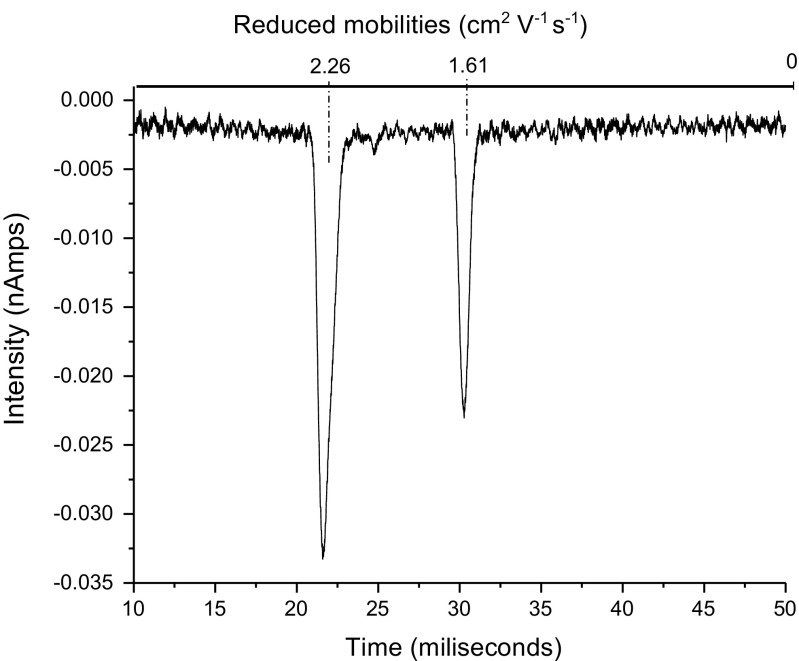
IMS spectrum of air doped with HCE with the addition of ENF

*ENF* Only ENF.Cl^–^ is observed – see Figure 7. This is consistent with the energetics given in Table 6. Again, the strain of an 8-membered ring compared with a 7-membered ring accounts for the instability of the complex with Cl_2_^–^.

**Table 6:** ΔHs and ΔGs for the Reaction of ENF with the HCE RIP. DFT Calculations Were Performed Using the B3LYP Functional and the 6-31 + G (d,p) Basis Set

Reactants	Products	ΔH_298_ kJ∙mol^–1^	ΔG_298_ kJ∙mol^–1^
ENF + Cl^–^	ENF.Cl^–^	–104	–70
ENF + Cl_2_^–^	ENF.Cl_2_^–^	–68	–25
ENF.Cl^–^ + ENF	ENF_2_.Cl^–^	–66	–33

**Figure 8:**
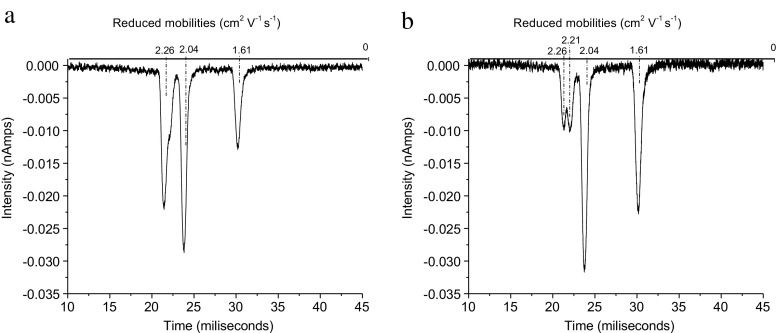
IMS spectra of air doped with enough HCE to have similar amounts of air RIP and HCE RIP after introducing a small amount of ENF (**a**) and a higher amount of ENF (**b**)

Again, it was subjectively observed that the air system was much less responsive to ENF than was the HCE system. Using the approach outlined for ISOF, the relative sensitivity to ENF in an air and HCE system was investigated – see Figure 8. When sufficient ENF to deplete the HCE peak by 50% was added, no appreciable diminution of the air RIP occurred. Increasing the ENF concentration to virtually remove the Cl^–^ RIP leaving just the Cl_2_^–^ peak caused a small diminution of the air RIP with a corresponding trace of the dimer. A serendipitous experiment occurred when at the end of an air/ENF experiment the system was allowed to diminish the ENF concentration and return to a good air RIP. On addition of HCE, a strong ENF.Cl^–^ peak appeared.

## Conclusions

ENF and ISOF do not produce Cl^–^ in an IMS system by capture of free electrons or reaction with O_2_^-^. This demonstrates that the Cl^–^ containing ions reported in the earlier study were the result of a chlorine containing contaminant as suggested [1]. The failure of ENF and ISOF to undergo DEA was initially surprising given the high calculated electron affinities, but further calculations showed that this was a result of the large positive VAEs. The present data are consistent with those of Matias et al. in their crossed electron-molecular beam two sector field mass spectrometer [20]. An unusual observation is that the observed monomer complexes of ISOF and ENF with O_2_^–^ are relatively unstable compared with the dimer complexes. This is in contrast to the more usual observation that monomer complexes are more stable than dimer complexes as shown, for example, with the Cl^–^ complexes of ISOF and ENF. DFT calculations do show that stable complexes of ISOF and ENF with O_2_^–^ are possible and are the likely precursors of the dimer complexes. That they are not seen is a consequence of the initial complexes being unstable, and conformational changes leading to ring formation (7 and 8, respectively, for ISOF and ENF) are required to form the stable monomer. The instability of the initial complexes require a high concentration of ISOF and ENF for them to be formed in appreciable amounts and thus the stable monomers, once formed, immediately react to form the dimers. This is consistent with the observed low sensitivity of an oxygen-based system to ISOF and ENF. Dissociative electron attachment to HCE produces primarily Cl^–^ with a small amount of Cl_2_^–^, whereas electron transfer from O_2_^–^ gives much more Cl_2_^–^. It is suggested that Cl^–^ can be produced from any conformation of HCE, whereas Cl_2_^–^ can only be produced from at least a partially eclipsed conformation and that reaction with O_2_^–^ promotes this through a transient complex with HCE.

## Electronic supplementary material

Below is the link to the electronic supplementary material.

ESM 1 (DOCX 1289 kb)
