# Supplementary material for: Ion Mobility Studies on the Negative Ion-Molecule Chemistry of Isoflurane and Enflurane
Source: J Am Soc Mass Spectrom. 2017 Feb 21;28(5):939–46. doi: 10.1007/s13361-017-1616-0 (PMC5393067; doi:10.1007/s13361-017-1616-0)
Supplement: Supplementary file 1 — (DOCX 1289 kb) [file 13361_2017_1616_MOESM1_ESM.docx]

**SUPPLEMENTARY MATERIAL**

**Ion mobility studies on the negative ion-molecule chemistry of isoflurane and enflurane**

Ramón González-Méndez,^1*^ Peter Watts,^1^ David C. Howse,^1^ Immacolata Procino,^2^ Henry McIntyre,^2^ and Chris A. Mayhew^1,3^

1. School of Physics and Astronomy, University of Birmingham, Edgbaston, Birmingham, B15 2TT, UK
2. Smiths Detection, Watford Ltd, Century House, Maylands Avenue, Hemel Hempstead, Hertfordshire, HP2 7DE, UK
3. Institut für Atemgasanalytik, Leopold-Franzens-Universität Innsbruck, Rathausplatz 4, A-6850 Dornbirn, Austria

* Corresponding Author Tel.:+44 121 414 4668. E-mail: [R.GonzalezMendez@bham.ac.uk](mailto:rR.GonzalezMendez@bham.ac.uk)

This supplementary material is formed by Figures S1 to S4.

**
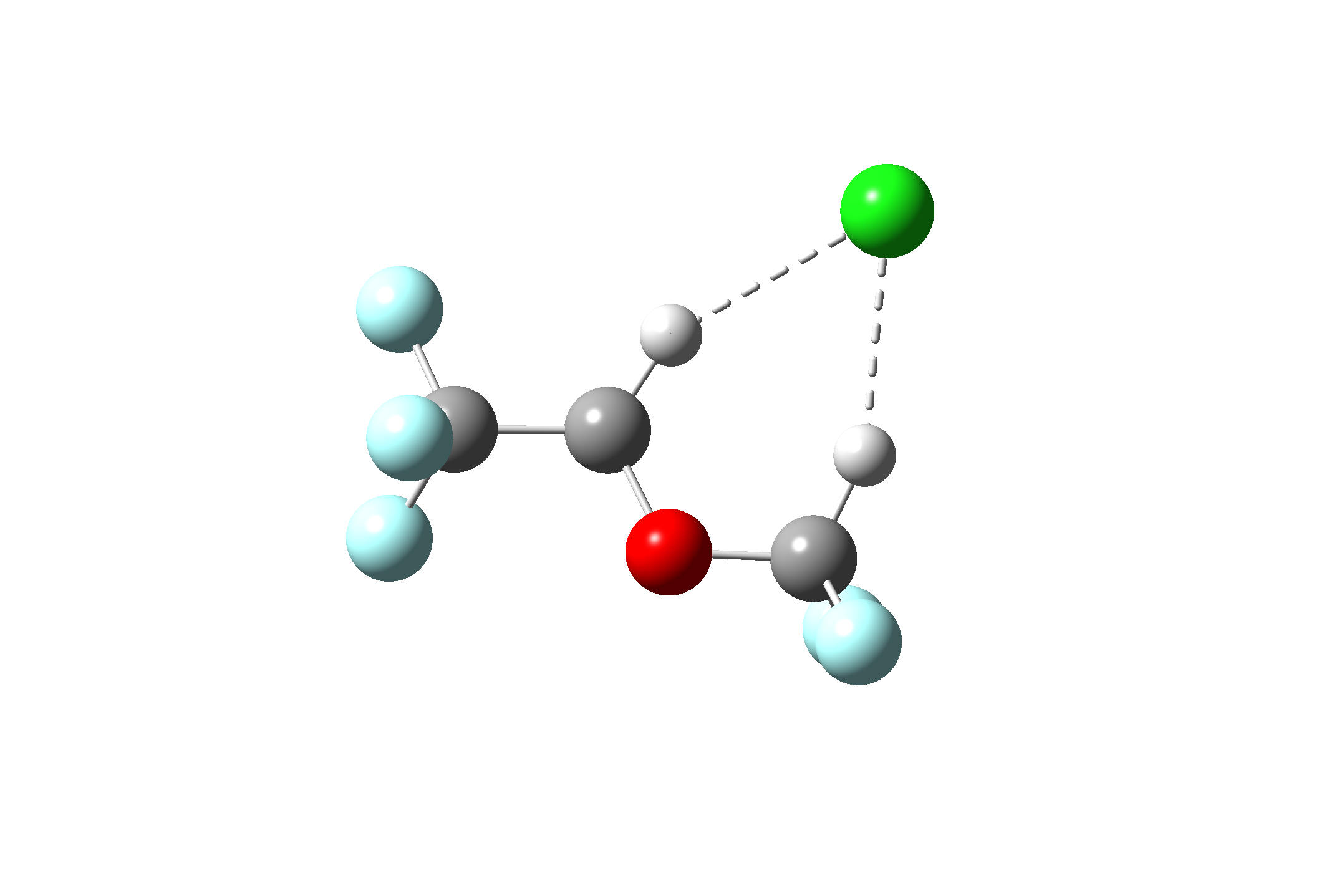
**

Figure S1. Structure of ISOF^-^ (CF_3_CHClOCHF_2_)^-^ obtained from DFT calculations.

| 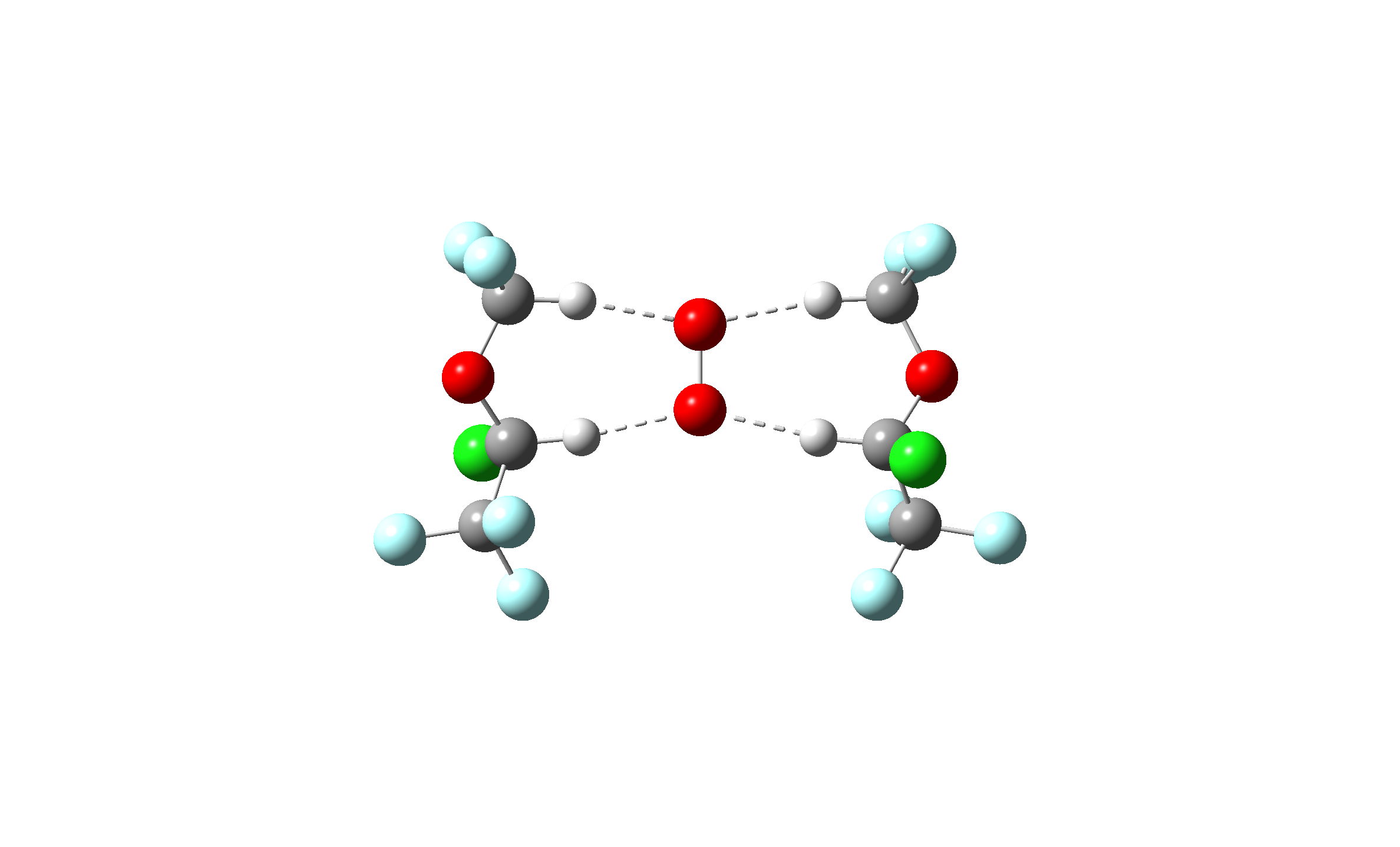 | 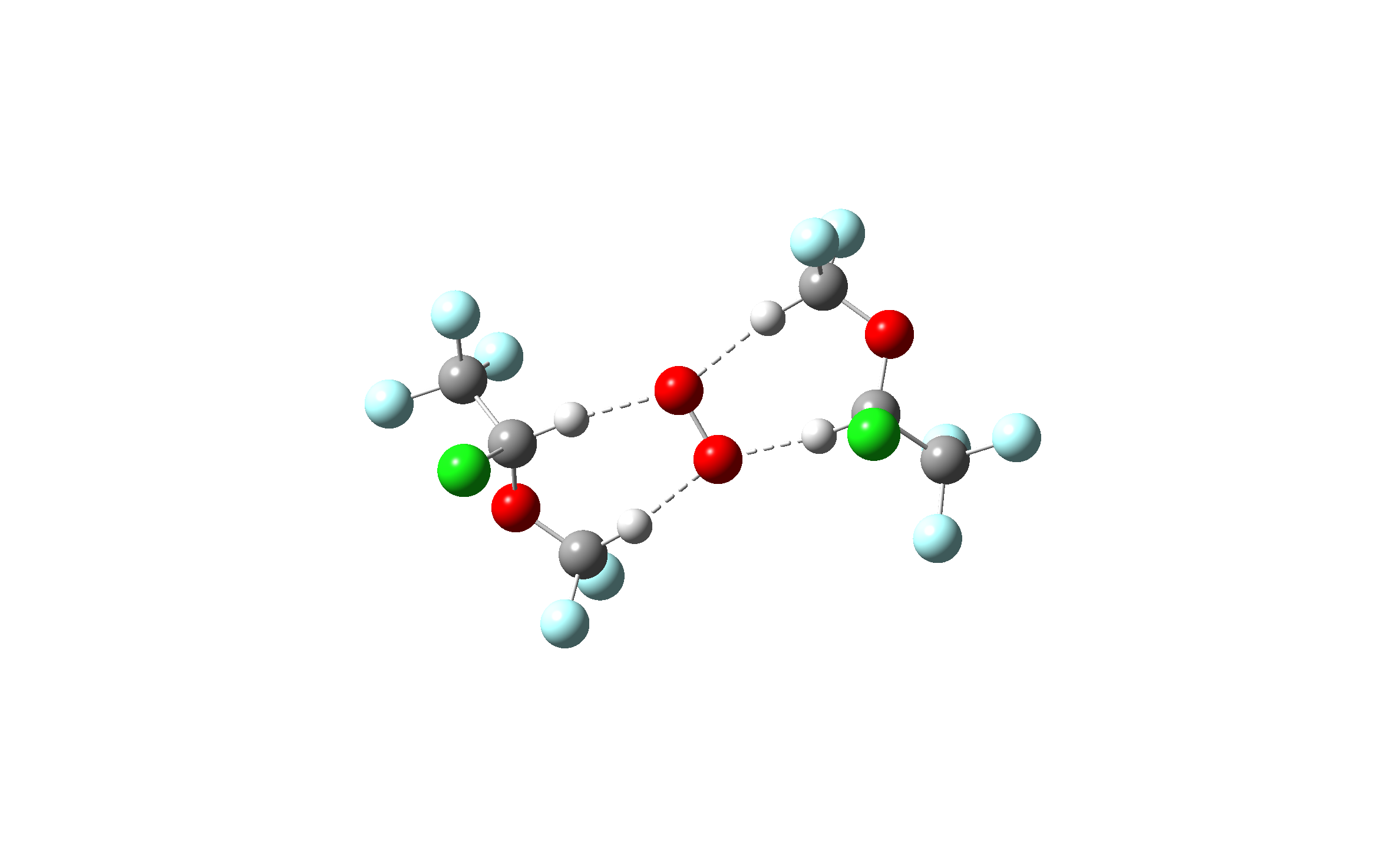 |
| --- | --- |
| (ISOF)_2_.O_2_^-^(anti) | ISOF_2_.O_2_^-^(syn) |

Figure S2. Two structures for the dimer ion (ISOF)_2_.O_2_^-^ from DFT calculations.

| 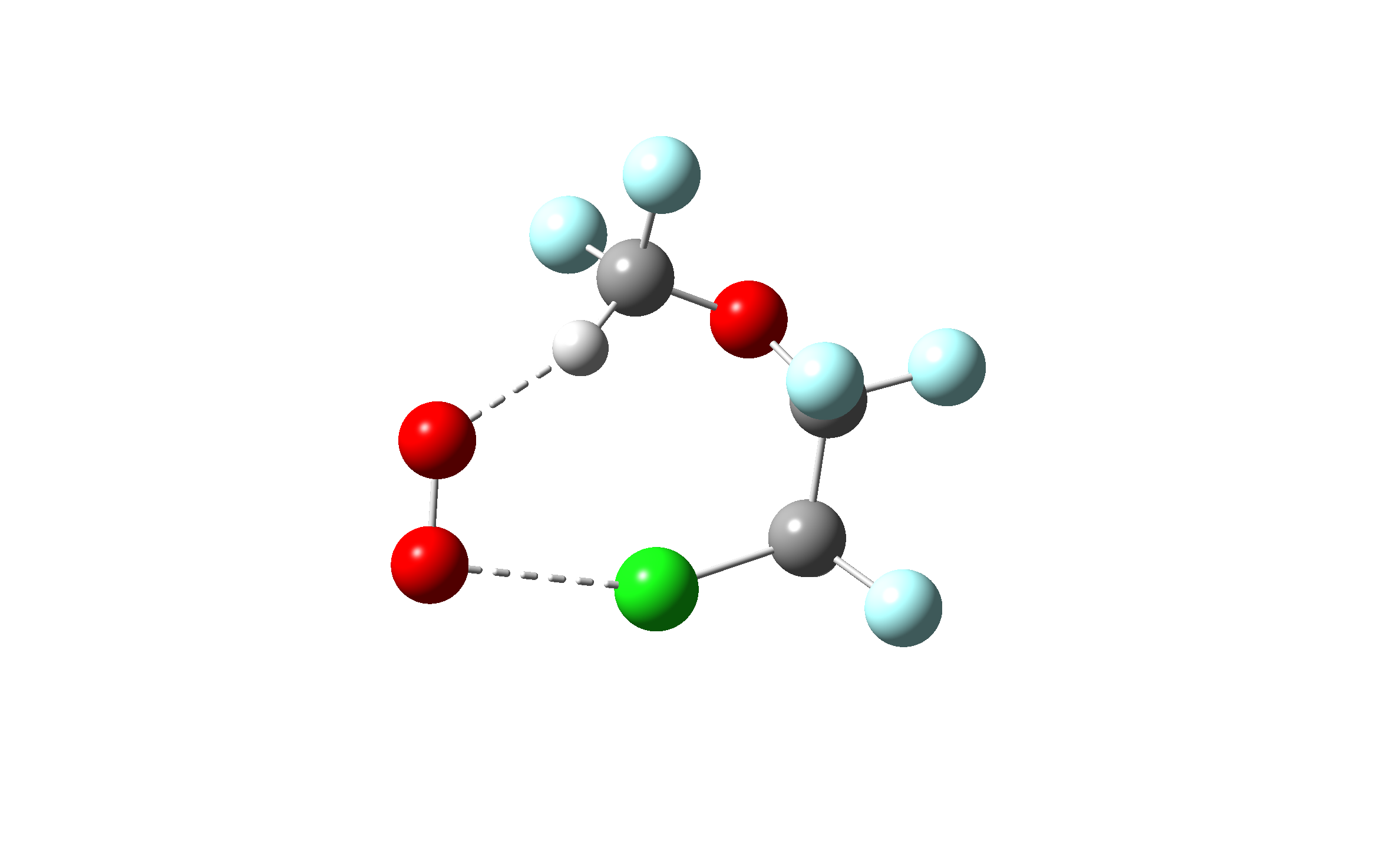 | 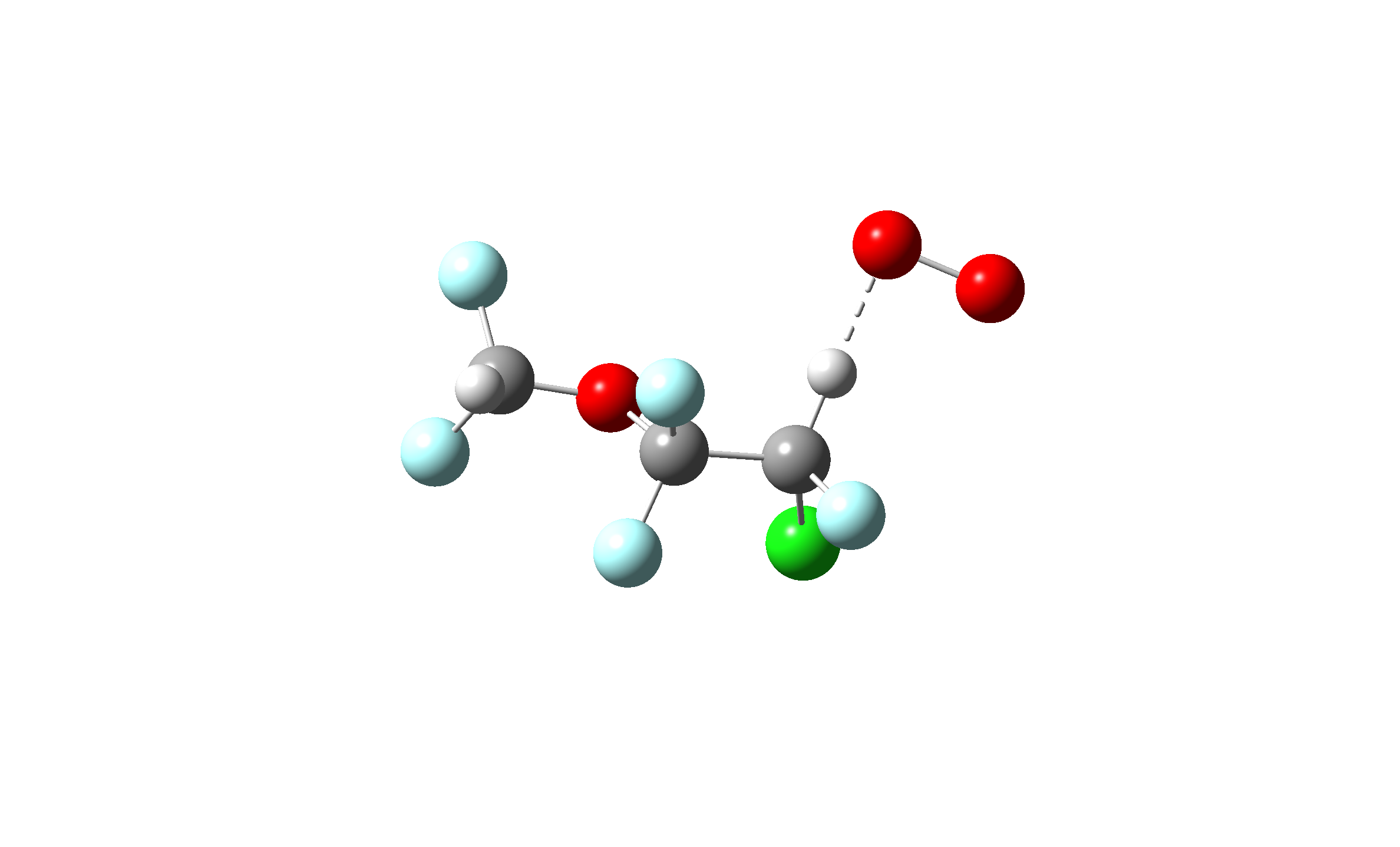 |
| --- | --- |
| ENF.O_2_^-^(a) | ENF.O_2_^-^(b) |
| 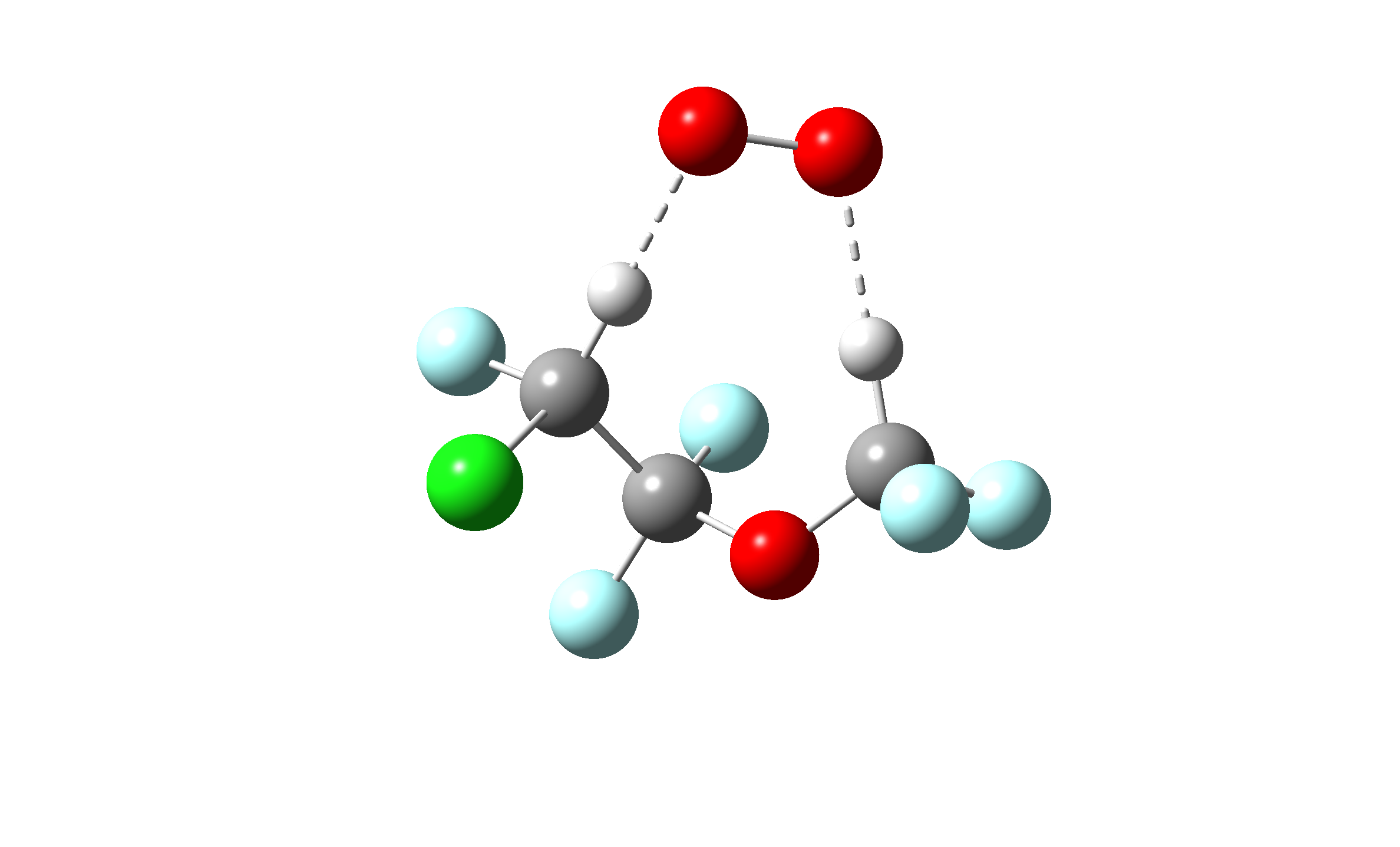 | |
| ENF.O_2_^-^(c) | |
| Figure S3. Three stable structures for the monomer ENF.O_2_^-^ obtained from DFT calculations. | |

| 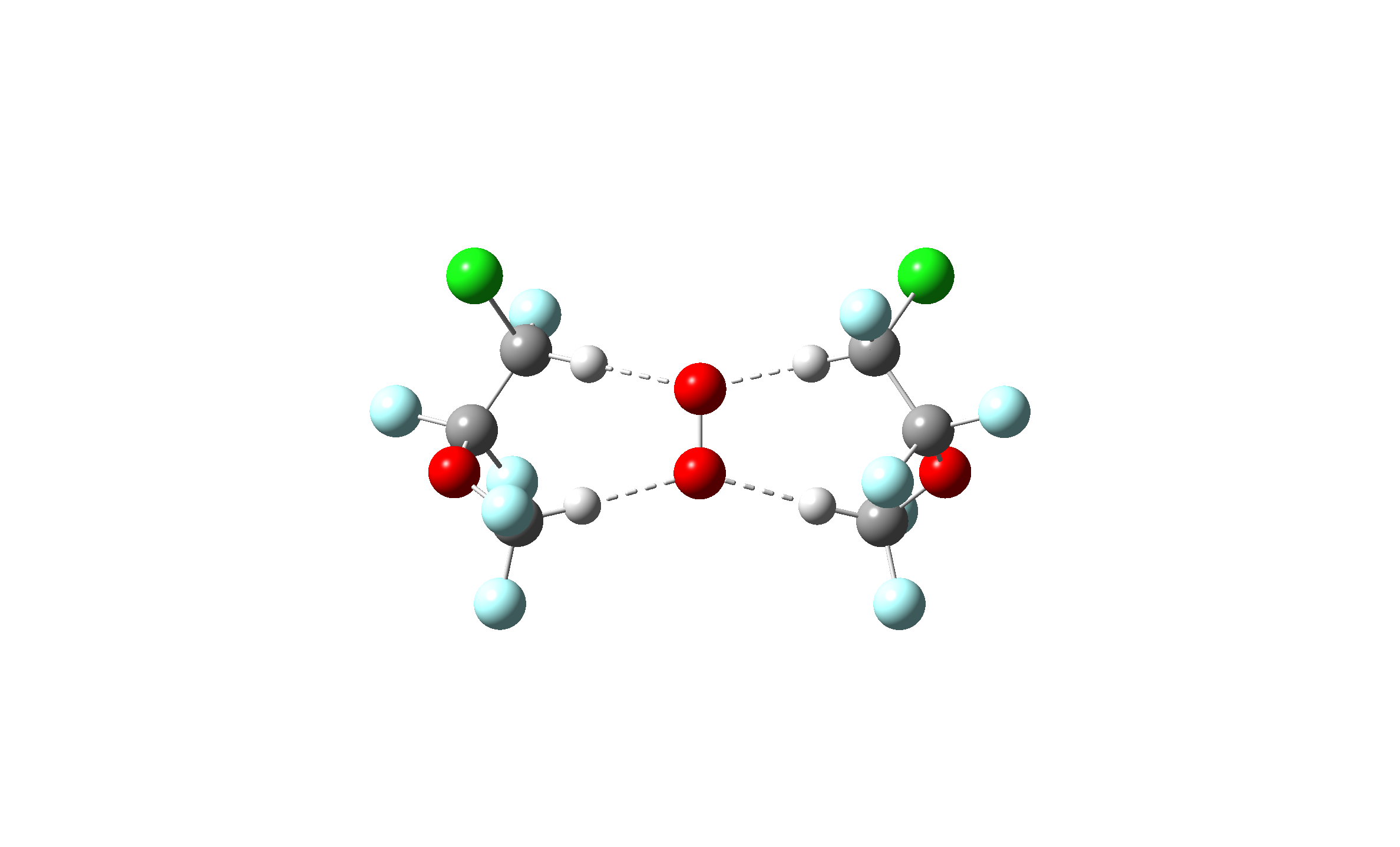 | 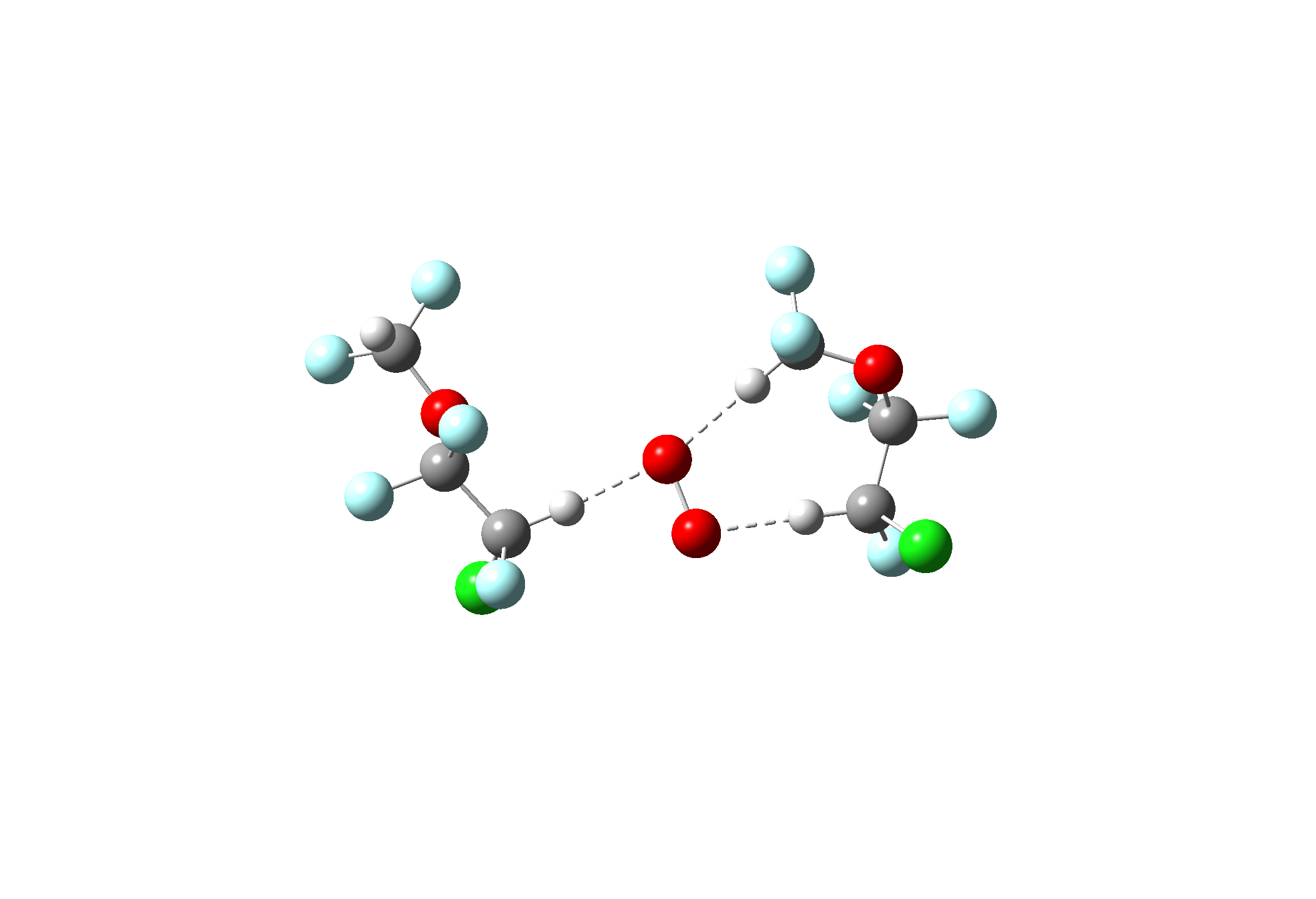 |
| --- | --- |
| (ENF)_2_.O_2_^-^(a) | (ENF)_2_.O_2_^-^(b) |

Figure S4. Two structures for (ENF)_2_.O_2_^-^ obtained from DFT calculations.
